# The Effect of Group Play Therapy on Social-Emotional Skills in Pre-School Children

**DOI:** 10.5539/gjhs.v6n2p163

**Published:** 2013-12-24

**Authors:** Ahdieh Chinekesh, Mehrnoush Kamalian, Masoumeh Eltemasi, Shirin Chinekesh, Manijeh Alavi

**Affiliations:** 1 Center for Development and Cooperation of Research and Technology, Undersecretary for Research and Technology, Ministry of Health and Medical Education, Tehran, Iran; 2 Shahid Beheshti University of Medical Sciences, Tehran, Iran

**Keywords:** play therapy, social-emotional skills, pre-school children, adaptive behaviors

## Abstract

**Background:**

Childhood is important and critical period in human life. The foundation of ego is shaped in childhood. Play therapy is one of the successful strategies to help children with inner conflicts problems. This method of psychotherapy is base on the normal learning processes of children, provides solutions to relieve feelings of stress, and expands self-expression. Group play therapy can enhance the self-awareness, self- regulation, social communication, empathy and adoptability in children.

**Methods:**

Present study investigated the effects of play therapy on relational and emotional skills of pre-school children. For this purpose, the total numbers of 372 pre-school children were randomly selected, and divided into two equal groups (case and control). In next step, the BUSSE-SR methodology was used for evaluation and comparison of self-awareness, self-regulation, social interaction, empathy, adoptability, and control groups. Pre-test were performed for both groups and case group was involved in-group play therapy. According to the results of post-test, correlation of variables between case-control groups was examined by multivariate analysis of covariance.

**Results:**

Frequency of boys and girls in our sample were 51.3 and 48.7 percent, respectively. The mean age of children was 5.1±0.6 year. According to the results of present study, play therapy significantly enhanced the social-emotional skills (P< 0.001). Our findings are consistent with the results of previous studies in other nations with different environmental and cultural properties. In conclusion, it seems that play therapy can be used in pre-school centers to help children learn problem-solving skills and communicate with others.

## 1. Introduction

Childhood is the important and critical period of life with critical effects on personality of individuals. In other hand, it is best period to help children learn adaptive behaviors and effective communicational skills (Kollbrunner & Seifert, 2013). In next periods of life, especially in of adolescence, these skills can provide corrective emotional strategies and healing in conflicts (Jager, 2013). Recently, effect of play therapy for improving of social skills has been noticed by most of the socio-science researchers (Stone & Stark, 2013). Many researchers believe that the lack of social-emotional skills educations considered as a reason for the failure of many children in school, it means that academic achievements need not only cognitive abilities but also social and emotional competence (Chari et al., 2013).

Social-emotional development includes a set of skills such as self-awareness, understanding of others emotions, emotional management, emotional expressions in a constructive manner, self-regulation, stabled communications (Abdollahian, Mokhber, Balaghi, & Moharrari, 2013). Social-emotional development is also important to achieve optimal growth in social, educational, and career (Ryan & Edge, 2012). Children with social-emotional skills are successfully preventing or resolving psychosocial difficulties, and effectively communicating both with adults and peer groups (Jafari, Mohammadi, Khanbani, Farid, & Chiti, 2011). Through play and play therapy, children can learn many basic social-emotional skills to communicate and express their feelings in relating to others (Rye, 2008).

Play therapy helps children with problems to provide a safe distance from their psychological problems and express appropriate interactions (Ryan & Edge, 2012; Jafari et al., 2011). Play therapy could be used as means of communication between child and therapist (Kool & Lawver, 2010). It helps child to come out from itself, communicates with the outside world, and interact with their environment (Rye, 2008; Lawver & Blankenship, 2008).

Knell (1998) used play therapy as educational tool in dealing with children problems and as alternative to verbal tools. Jafari et al. (2011) assessed the effect of play therapy on behavioral problems of mal-adjusted pre-school children. The results showed significant difference in mean score of post-tests of case and control groups, and deficit of attention in hyperactive children was reduced after play therapy. They also found that play therapy has significantly affected on behavioral problems in children who received intervention. Hatami, Yousefi and Delavar (2012) reported the impact of group games and play therapy on behavioral disorders in children. They showed the positive correlation between play therapy and reduction of communication disorders.

In present study, we examined the impact of group play therapy on self-awareness, self-regulation, social interaction, empathy, and adoptability of pre-school children in Tehran, Iran.

## 2. Material and Methods

In present study, we used pre and post-test methodology for comparison the differences of self-awareness, self-regulation, social interaction, empathy, and adoptability between case and control groups. According to the number of pre-school children in the area understudy in Tehran (11697) and based on Morgan table, a random distributed population including 372 individuals were selected. We divided sample population randomly in two equal groups including case and control (186 in each group). In next step, BUSSE-SR social-emotional questionnaire was used (Miller, Johnston, Klassen, Fine, & Papsdorf, 2005). The questionnaire included 50 questions that measures self-awareness, self-regulation, social communication, empathy and adoptive behavior. The questions were simple and clear and its reliability and stability previously have been approved (Miller et al., 2005).

In first step, we examined both groups by pretest. For this purpose, parents of selected children filled the questionnaire according to the instructions to fill. We collected questionnaires after 2 days and analyzed. The case group was involved in directed social-emotional play therapy during the fifteen sessions of 90 min (Three sessions a week). In directed play therapy, the therapist was responsible for selection and leadership of the games. Children were encouraged to express their problems and find solutions. Then, the post-test was carried out for both groups by written. We performed analysis of covariance (ANCOVA) by SPSS 17 to compare mean results and control groups.

## 3. Results

According to the findings of this study, 51.3and 48.7 percent of children were boys and girls respectively. The mean of age was 5.1years old. The mean of height was 108.3 centimeters for girls and 110.1 centimeters for boys. In addition, the mean of weight was 17.3 kg for girls and 18.9 kg for boys. All the children were mentally and physically normal. We did not find any significant differences in mean scores of two groups in pre-test (Table 1).

**Table 1:** Comparison of mean scores of social-emotional skills of case and control groups in pre-test

Variable	Group	Mean Score	Standard Error	Error of the Mean
**Self-Awareness**	Control	2.425	0.451	0.033
	Intervention	2.381	0.4	0.029
**Self-Regulation**	Control	2.404	0.34	0.024
	Intervention	2.411	0.327	0.024
**Empathy**	Control	2.407	0.87	0.063
	Intervention	2.405	0.92	0.067
**Social Interaction**	Control	2.41	0.336	0.024
	Intervention	2.412	0.34	0.025
**Adoptability**	Control	2.385	0.419	0.03
	Intervention	2.356	0.47	0.034
**Social-emotional skills**	Control	2.407	0.183	0.013
	Intervention	2.398	0.183	0.013

As we can see in table 1, the mean scores of two groups in pre-test are closed. The comparison of mean scores in control group in pre-test and post-test has been shown in Table 2. There were no considerable differences between mean scores in pretest and post- test in control group. Table 3, shows that the mean scores of all variables in post-test has been increased in case group. In the other words, group playing had positive effects on scores. By multivariate analysis of covariance (ANOVA) we found that the mean score of the control group had no significant changes in self-awareness (P >0.05) whereas awareness mean score in intervention group has significantly increased. (P <0.01 one-way) Self-regulation mean score in control group showed no significant changes in post-test (P >0.05), but in case group, significant changes in self-regulation scores was detected (P <0.01 - one-way). In addition, in other variables (social interaction, empathy, adoptability) mean scores of case group significantly increased (P <0.01 - one tail) (Tables 2 & 3). According to the descriptive and analytical results of this study, play therapy significantly improved social-emotional competence of samples.

**Table 2:** Pre-test and post-test mean score differences of socio-emotional skills in control group

Variable	Group	Mean Score	Standard Error	Error of the Mean
**Self-Awareness**	Pretest	2.425	0.451	0.033
	Post test	2.398	0.41	0.03
**Self-Regulation**	Pretest	2.404	0.34	0.024
	Post test	2.395	0.364	0.026
**Empathy**	Pretest	2.407	0.87	0.063
	Post test	2.392	0.95	0.069
**Social Interaction**	Pretest	2.41	0.336	0.024
	Post test	2.37	0.346	0.025
**Adoptability**	Pretest	2.385	0.419	0.03
	Post test	2.334	0.486	0.035
**Social-Emotional Skills**	Pretest	2.407	0.183	0.013
	Post test	2.386	0.186	0.013

**Table 3:** Pre-test and post-test mean score differences of social-emotional skills in case group

Variable	Group	Mean Score	Standard Error	Error of the Mean
**Self-Awareness**	Pretest	2.381	0.4003	0.029
	Post test	3.619	0.4007	0.029
**Self-Regulation**	Pretest	2.411	0.328	0.024
	Post test	3.568	0.363	0.026
**Empathy**	Pretest	2.405	0.921	0.067
	Post test	3.661	0.887	0.065
**Social Interaction**	Pretest	2.412	0.344	0.025
	Post test	3.567	0.329	0.024
**Adoptability**	Pretest	2.356	0.473	0.034
	Post test	3.604	0.468	0.034
**Social-Emotional Skills**	Pretest	2.398	0.183	0.013
	Post test	3.588	0.188	0.013

## 4. Discussion

In present study, we considered the impact of directed group play therapy on improvement of social-emotional skills of pre-school children. Our hypothesis was the play therapy method could be an effective and general approach in training of children to establish communications, express thoughts and feelings, and solve their problems (Rye, 2008; Jager & Ryan, 2007). We studied self- awareness, self-regulation, social interaction, empathy, and adoptability as components of social-emotional skills (Dvarionas, 2002). Our study revealed group game therapy significantly improves cognitive development. Our findings were conformed with the results of previous studies about the roles of group play therapy on behavioral and emotional performance of children (Jager & Ryan, 2007; Hill, 2009; van Breemen, 2009).

The efficacy of group play therapy has been considered in some studies, and they indicated that this method is effective to prevent or resolve psychosocial problems (Hubbard, 1991; Johnson, 1988; VanScoy, 1972). For instance, Reams and Friedrich (1994) found play therapy has increased social development of children. Similarly, Pepin and Stagnitti in 2012 showed that play therapy had positive effects on Creativity of intervened children. In several independent studies, authors found that play therapy promotes self-confidence and reduces anxiety and depression (Mizuno & Sakuma, 2013; Seifert & Kollbrunner, 2005; Kuipers & Clemens, 1998).

Panksepp, Burgdorf, Turner and Gordon (2003) have reported the directed group games can reduce behavioral disorders in primary school students. Venker, McDuffie, Ellis Weismer and Abbeduto (2012) found that Play therapy reduced parent-child relationship stress. Hunter (1993) showed that play therapy had positive effects on developmental and diagnostic factors in children who were homeless. Although, most of these studies have assessed the role of play therapy in treatment of psychological disorders, some studies showed the game therapy also could be effective in reduction of normal children social problems (Panksepp et al., 2003; Lewis, 1993; Moustakas, 1951).

## 5. Conclusion

Considering the results of present study and other research results, it can be concluded that play therapy provides environment where children can measure their own abilities, express themselves, and learn how to use their knowledge to take maximum advantages of their capacities. Finally, to extend the application of group game therapy method benefits and enhance children’s social-emotional skills training of related skills to teachers in childhood education centers are recommended.
